# Subtypes of Alzheimer’s disease: questions, controversy, and meaning

**DOI:** 10.1016/j.tins.2022.02.001

**Published:** 2022-02-25

**Authors:** Jacob W. Vogel, Oskar Hansson

**Affiliations:** 1 Penn/CHOP Lifespan Brain Institute, University of Pennsylvania, Philadelphia, PA 19104, USA; 2 Department of Psychiatry, University of Pennsylvania, Philadelphia, PA 19104, USA; 3 Clinical Memory Research Unit, Department of Clinical Sciences Malmö, Lund University, Sweden; 4 Memory Clinic, Skåne University Hospital, Malmö, Sweden

## Abstract

The distribution of Alzheimer’s disease (AD) tau pathology varies systematically and causes a diverse array of syndromes. This forum article provides a brief overview of key controversies in untangling the complexity of AD subtypes, explores potential causes of AD variability in the population, and discusses clinical relevance and future directions of research into AD heterogeneity.

## The landscape of clinical heterogeneity in AD

AD is characterized by the pathological accumulation of amyloid-*β* plaques and neurofibrillary tau tangles in the brain. Traditionally, the disease is associated with a slowly developing late-life syndrome, first involving memory impairment, followed by multidomain cognitive impairment, and eventually frank dementia. However, several syndromes have been described that involve initial and predominant impairment in either visuospatial cognition, language production, executive function, personality, and behavior or motor faculties, which nonetheless feature AD as the primary pathology [1]. The age of onset of these nonamnestic AD cases is often younger than that of amnestic AD, often beginning before the age of 65 years.

The contrast between amnestic and nonamnestic clinical presentations is mirrored by differences in the radiological presentations of AD variants. The gradual cognitive decline of late-onset amnestic AD is accompanied by progressive neurodegeneration, predominantly of medial temporal lobe structures and, later, posterior transmodal association cortex. Meanwhile, young onset and nonamnestic clinical presentations of AD frequently feature tau accumulation and neurodegeneration in regions highly specific to the domain impairment, often with a relative sparing of hippocampus and medial temporal structures vulnerable in amnestic AD [1]. The distribution of amyloid-*β* does not vary strongly across different AD phenotypes, but tau patterns align closely with clinical presentation [2].

**Figure 1:**
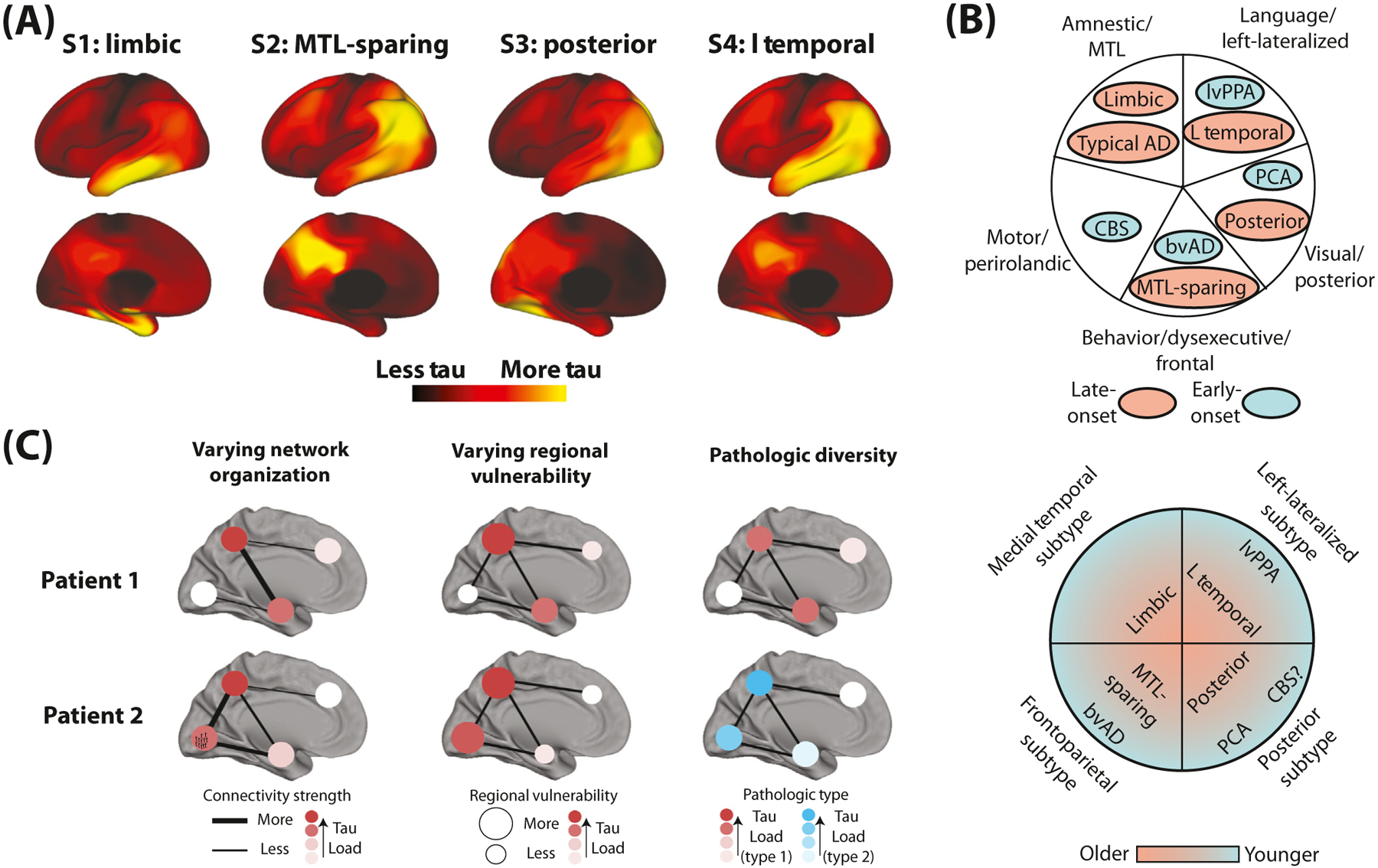
Conceptualizing the classification and potential source of variation in AD tau pathology. (A) Four subtypes of tau distribution derived using spatiotemporal disease progression modeling, as described in [6]. (B) Top: a view of AD phenotypes where ‘atypical’ and late-onset AD presentations share certain qualities but are nonetheless separate entities. Bottom: a view that places AD phenotypes along four different age-associated spectra, such that ‘atypical’ clinical variants are extreme, early-onset expressions of common AD phenotypes. (C) Three different scenarios that could lead to distinct expression patterns of AD pathology across individuals. Left: variation in axonal connectivity patterns results in pathology or pathological states accumulating in a pattern determined by individual network architecture. Middle: certain vulnerable regions accumulate pathology at a greater rate in certain individuals, leading to variation in secondary epicenters. Right: different instances of AD pathology exist featuring distinct biochemical or biophysical profiles causing differential regional spread and expression or spread properties. Abbreviations: AD, Alzheimer’s disease; bvAD, behavioral variant AD; CBS, corticobasal syndrome; L temporal, lateral temporal; lvPPA, logopenic variant primary progressive aphasia; MTL, medial temporal lobe; PCA, posterior cortical atrophy.

The observation of AD variants with an (often) early age of onset and profound initial deficits in nonmemory domains has prompted many researchers to label these syndromes as ‘atypical AD’, to contrast them against ‘typical’ late-onset amnestic presentations. However, recent work suggests greater variation in the clinical presentation of late-onset AD than has previously been appreciated. Late-onset cases with especially strong deficits in non-memory domains are not uncommon [3]. Additionally, an important autopsy study found systematic variation in the relative regional distribution of tau tangles within a large sample of AD patients [4]. A limbic-predominant and a hippocampalsparing phenotype were described in contrast to a ‘typical’ AD pattern (involving both limbic and cortical tau accumulation). These tau variants exhibited distinct demographic, genetic, and cognitive profiles from one another and from other forms of limbic tauopathy [4,5]. Our group recently confirmed these phenotypes *in vivo* using a multisite dataset of over 2000 tau-positron emission tomography scans, and we additionally described the existence of a posterior and an asymmetric lateral temporal pattern of tau accumulation [6] (Figure 1A). Patterns similar to these latter phenotypes have been described in studies probing neurodegenerative profiles of late-onset AD cases with impairments in specific cognitive domains [3]. In our study, we did not find one dominant tau pattern that fits the description of ‘typical’ AD. Rather, all four AD tau subtypes exhibited some degree of memory impairment and ‘typical’ atrophy patterns occurred at different timepoints along each subtype’s trajectory [6]. The study adds to the growing literature underscoring the heterogeneity of AD, although several critical knowledge gaps remain. This forum article discusses how heterogeneity in the accumulation of tau relates to atypical clinical variants, what factors might cause it, and what it means for clinical research and practice.

## Atypical phenotypes: distinct entities or the extremes of spectra?

Many of the tau subtypes mentioned earlier, despite being evident in both early- and late-onset patients, strongly resemble previously described nonamnestic clinicoradiological syndromes. Indeed, direct comparisons of the atrophy patterns of atypical clinical variants and cognitively defined late-onset subtypes show a remarkable spatial correlation [3]. At present, whether these phenotypes represent two distinct entities or two points along a continuum remains unclear (Figure 1B). Advances in imaging, histology, and bioinformatics may yet reveal features that readily distinguish young-onset, nonamnestic AD variants from their homologous late-onset counterparts. Perhaps these homologous variants result from distinct etiological events, which nonetheless converge in selectively targeting certain networks of vulnerable brain regions. However, the observation that tau can deposit selectively in certain brain regions in certain patients, no matter their age of onset, indicates some level of shared vulnerability among these groups of patients. Investigating subtypes of AD based on biology (e.g., tau pathology) rather than clinical symptomatic expression may therefore be an important consideration in the quest to understand AD neurobiology.

## Possible sources of variation in the expression of AD

Little is known about why a single diagnostic entity can result in such variation in regional expression, symptoms, and severity. One study showed biochemical characteristics, such as site of tau phosphorylation, contribute substantially to variation in rate of tau seeding, correlate with rate of cognitive decline, and inversely correlate with age [7]. Importantly, the rate (and type) of tau seed formation of fibrils in individual patients correlated with the rate of seed formation when those fibrils were injected into mice, indicating increased production was an intrinsic feature of the fibril rather than its environment. Individual variation in sleep may also affect the production of tau pathology, perhaps through moderating neural activity [8,9]. Population variance in clearance of pathologic proteins is another potential culprit for age-related variation in rate of disease progression. Given the same rate of production, genetic or environmental vulnerability of endosomal/lysosomal systems could result in a faster (i.e., earlier in life) arrival at pathological accumulation of misfolded amyloid-*β* and tau oligomers, and a protracted accumulation period due to failure of degradation and clearance mechanisms [10]. Larger genome-wide association studies may provide additional insight into factors relating to the rate and severity of AD progression.

A fundamental unanswered question pertains to whether diversity in AD expression is due to distinct disease mechanisms or basic population variation. Like other neurodegenerative diseases, AD pathology can selectively manifest (initially) in different behavioral/cognitive systems [11]. This is consistent with the idea that such systems are distinguished through intrinsic properties such as anatomical network community and/or cell type specificity. What is unclear, then, is whether the diverse expression and spread of AD tau pathology represents an essentially uniform pathology, the distribution of which varies based on: (i) individual differences in brain organization, or (ii) regional vulnerability. Alternatively, variation may be caused by (iii) intrinsic properties of disease pathology dictating the pattern of expression (Figure 1C). These scenarios are laid out in greater detail in Box 1. Interestingly, a recent twin study suggested the genome plays a large role in individual tau patterns, while environmental factors may moderate disease timelines [12].

## Concluding remarks and future directions

Age of onset and AD subtype are both relevant to the rate of longitudinal decline [6], but further research is necessary to translate distinct manifestations of AD into insights for clinical management, prognosis, or treatment. These efforts would be accelerated by consensus on the definition and formal identification of subtypes. Much work is also needed to better characterize variation in the expression of AD. This would include surveying functional neuroimaging, cerebrospinal fluid profiles, and multi-omic regional tissue profiles of individuals across different AD subtypes. Further, variation in the expression of AD (and other neurodegenerative diseases) appears somewhat constrained by selective vulnerability to intrinsic functional-behavioral networks [11] and biological substrates of such networks should continue to be better characterized. Insofar as subtypes of AD reflect distinct underlying disease biology, *a priori* or *post hoc* identification of subtypes may help reveal groups of patients that respond better or worse to different interventions, hopefully leading to more personalized treatment.

While idiosyncratic differences in disease expression surely exist at a higher resolution of investigation, variation in the expression of AD appears to be somewhat systematic. Accumulating evidence reviewed here points to subtypes of AD that capture internally consistent patterns of population variation that are otherwise distinct from other manifestations of AD. This observation bodes well for the discovery of sources of this variation, as such sources may occur somewhat regularly in the population, suggestive of effect sizes within range of detection. An example might be the recurrent finding that carriage of the APOE4 allele is associated with a limbic-amnestic expression of AD [4,6,15]. Therefore, studying subtypes may serve as a stepping stone toward individualized treatment for AD.

## Box 1. Potential causes of AD subtypes

- Variation in brain organization. A biochemical process consistent across individuals initializes the spread of pathology, but the epicenter varies due to systematic population differences in brain organization. This individual network vulnerability could be the result of longterm accumulation of metabolic strain, as might be suggested by the greater incidence of premorbid language learning disabilities in logopenic variant primary progressive aphasia patients [13]. Population variation in network organization might facilitate selective spread to/through certain networks. Such variation could be detectable through premorbid (i.e., population variation in) functional or structural neuroimaging and may implicate network-modifying therapies.
- Variation in regional vulnerability. The initial pathological cascade may not vary between individuals, but different regions may become particularly burdened in specific vulnerable brain regions along the cascade. The prominence of pathological burden in one region over another may then have a downstream effect in leading to a distinct progression. Supporting this theory, simulation models suggest tau subtypes involve deposition along different temporal lobe networks, driven by selective aggregation and spread from distinct secondary epicenters [6]. Many factors might lead to variation in individual-level regional vulnerability, including differences in cell type vulnerability, vascularization, oxidative stress, clearance processes, or inflammatory response. Note that this scenario is not mutually exclusive to scenario (i).
- Pathologic diversity. Variation in the biochemistry of disease pathology may exist in the population and such diversity might dictate how the disease manifests. Some precedence for this idea exists; differently conformed synthetic or patient-derived tau oligomers displayed different regional expression patterns when injected into mouse models of AD [7,14]. Heretofore unexplored individual differences in enzymatic activity, co-pathology, or other biological processes might lead to variation in pathologic diversity. In such cases, one type of treatment may not sufficiently target all expressions of AD pathology.
